# LncRNA CASC11 promotes the cervical cancer progression by activating Wnt/beta-catenin signaling pathway

**DOI:** 10.1186/s40659-019-0240-9

**Published:** 2019-06-29

**Authors:** Wenchan Hsu, Lifen Liu, Xin Chen, Ying Zhang, Weipei Zhu

**Affiliations:** 1 Department of Gynecology and Obstetrics, The Second Affiliated Hospital of Soochow University, Suzhou, 215004 China; 2 Department of Gynecology and Obstetrics, Xiamen Chang Gung Hospital, Xiamen, 361000 China

**Keywords:** LncRNA, *CASC11*, Cervical cancer, Wnt/β-catenin

## Abstract

**Background:**

Studies have shown that cancer susceptibility candidate 11 (*CASC11*), a newly discovered long non-coding RNA (lncRNA), was aberrantly overexpressed in hepatic carcinoma, gastric cancer and colorectal cancer. However, its effects on cervical cancer has been kept unknown up to now. The present study was aimed to investigate the relationship between lncRNA *CASC11* and cervical cancer and further explore the mechanism of *CASC11* effect on cervical cancer progression.

**Materials:**

Quantitative real-time polymerase chain reaction (RT-qPCR) was used to detect the expressions of *CASC11* in cancerous and adjacent normal tissues of patients with cervical cancer as well as in cell lines. The proliferation, migration, invasion and apoptosis were assayed after transfecting the cell with si-CASC11 or pcDNA3.1-CASC11. TOP/FOP-Flash luciferase reporter assay and western blot were used to analysis the activation of Wnt/β-catenin signaling pathway. Si-CASC11-transfected HeLa cells were subcutaneously inoculated into male athymic (nude) mice to investigate the effect of *CASC11* on the tumor formation.

**Results:**

We discovered that *CASC11*, the expression of which was positively associated with the tumor size and the FIGO staging and negatively related to the patients’ survival rate, was up-regulated in the cervical cancer tissues and cell lines. Silencing *CASC11* inhibited the proliferation, migration as well as invasion and promoted the cell apoptosis. Conversely, overexpression of *CASC11* facilitated the cancer cell’s proliferation, migration and invasion ability and suppressed the apoptosis. Further study showed that *CASC11* promoted the migration and invasion of cervical cancer cells by activating Wnt/β-catenin signaling pathway and silencing *CASC11* inhibited the tumor growth in vivo.

**Conclusion:**

Our study demonstrated that *CASC11* promoted the cervical cancer progression by activating Wnt/β-catenin signaling pathway for the first time, which provides a new target or a potential diagnostic biomarker of the treatment for cervical cancer.

**Electronic supplementary material:**

The online version of this article (10.1186/s40659-019-0240-9) contains supplementary material, which is available to authorized users.

## Background

Cervical cancer is one of the most common malignant tumors in women worldwide with high morbidity and mortality. Numbers of factors, such as genetic influences and viral infection, are involved in this complex disease. It is well known that cervical cancer is developed from cervical intraepithelial neoplasia (CIN), in which the infection of human papillomavirus (HPV) plays an initial role in the progression of the cervical lesions [1]. However, studies have shown HPV infection alone is not a sufficient condition for cervical cancer [2]. Specific chromosomal changes mainly including abnormal amplifications of chromosome 3q and protooncogene MYC (8q24) also occurred in malignant cervical lesions [3]. Meanwhile, recent researches showed that there may exist a close relationship between HPV protein and long non-coding RNA (lncRNA) in cervical cancer [4]. Unfortunately, the pathogenesis of cervical cancer has not been fully understood up to now.

LncRNA is a class of single-stranded RNAs with a length of more than 200 nucleotides (nt) and without the capacity of coding proteins. It works in cell growth, survival, migration, invasion, differentiation, and other cellular processes. The abnormal expression or dysfunction of lncRNA is closely related to the occurrence of cancers [5]. With the development of molecular biology technology, many lncRNAs were found to be served as potential and minimally invasive diagnostic/prognostic biomarkers, and also as therapeutic targets for cancers. For example, lncPCA3 has been routinely used for the diagnosis of prostate cancer in clinic [6]. More than a dozen of lncRNAs including HOX transcript antisense RNA (*HOTAIR*), metastasis associated lung adenocarcinoma transcript 1 (*MALAT1*), colon cancer-associated transcript 2 (*CCAT2*) and so on, are involved in cervical cancer by interacting with proteins, miRNAs and other molecules, providing accurate targets for the treatment of cervical cancer patients [7].

In recent years, researchers have found a variety of cancer-related lncRNAs localized on chromosome 8q24. *CCAT2*, which was located on 8q24.21 and originally identified as an oncogenic lncRNA in colorectal cancer, was significantly up-regulated in many kinds of malignant tumors containing lung cancer, colorectal cancer, breast cancer, gastric cancer, cervical cancer and so on, indicating that *CCAT2* might be a therapeutic target [8]. Cancer susceptibility candidate 11 (*CASC11*), which was also located on 8q24.21 region, not only promoted the growth and metastasis of colorectal cancer cells by interacting with heterogeneous ribonucleoprotein K (hnRNP-K) and activating Wnt/β-catenin pathway [9], but also facilitated the cell proliferation, migration and invasion in hepatocellular carcinoma and in gastric cancer via activating PI3K/AKT signaling pathway and regulating cell cycle pathway, respectively [10, 11]. However, no one has yet reported the effects of lncRNA *CASC11* in cervical cancer.

Wnt/β-catenin pathway is a classical signaling pathway involved in the development and progression of cancers. It initiates the transcription of the downstream target genes through activating β-catenin. Studies have shown that the aberrant activation of the Wnt/β-catenin pathway is closely related to HPV-infected cervical cancer [12]. In the present study, we explored the relationship between lncRNA *CASC11* and cervical cancer finding that high expression of lncRNA *CASC11* promoted the growth and metastasis of cervical cancer through Wnt/β-catenin pathway, which provided a new target for the treatment of cervical cancer.

## Materials and methods

### Tissue samples collection

Cancerous and adjacent normal tissues of 50 patients with cervical cancer who had not received prior treatment were obtained from the Second Affiliated Hospital of Soochow University from 2015 to 2018. All patients signed the informed consent. The samples were immediately frozen in liquid nitrogen and stored at -80 °C. The pathological classification and clinical stages were performed to the International Federation of Gynecology and Obstetrics (FIGO) criteria. All patients signed the informed content. All the protocols in this study were approved by the Ethics Committee of the Second Affiliated Hospital of Soochow hospital.

### Cell culture and processing

Human foreskin keratinocytes (HEKn, Cat. no. C-001-5C) were obtained from the Cascade BiologicsTm (Portland, OR, USA) and cultured in EpiLife medium (Gibco/LifeTechnologies, Waltham, MA; Cat. No. M-EPI-500-CA). The human cervical cancer cell lines (HeLa, CaSki, SiHa, C-33A and MS751) were purchased from Cell Bank of the Chinese Academy of Science (Shanghai, China) maintaining in RPMI1640 medium. All the mediums were supplemented with 10% fetal bovine serum (FBS; Hyclone; Invitrogen, Camarillo, CA, USA) and 100 U/ml penicillin/100 µg/ml streptomycin (Invitrogen, Carlsbad, CA, USA) at 37 °C with 5% CO_2_. For the cell processing, 10 mM LiCl or 100 ng/ml Dkk-1, which are served as the Wnt signaling pathway activator and inhibitor respectively, were added in to the medium 36 h after cell transfection.

### Subcutaneous xenografts in mice

As previously described [13], 4 × 10^6^ HeLa cells, which were transfected with si-CASC11 and si-NC for 24 h, were subcutaneously inoculated into male athymic nude mice (n = 12, 6–8 weeks old). The tumors’ size was measured every 5 days with calipers and the volume of the tumors were calculated as length × (width^2^)/2. 35 days after affections, the tumors were removed surgically and measured the weight.

### Quantitative real-time polymerase chain reaction (RT-qPCR)

According to the manufacturer’s instructions, total RNA was extracted from cells with RNAiso Plus (Code No. 9108, TaKaRa, Dalian, China). According to the protocols of manufacturer, RNA quality was assessed using a NanoDrop 2000 Spectrophotometer (Thermo Scientific). Then 1 µg total RNA was converted into the first strand cDNA using PrimeScript RT reagent kit (Takara, Tokyo, Japan). Finally, the PCR amplification was performed using a SYBR^®^ Premix Ex Taq™ II kit (Code No. RR820A, TaKaRa). The reaction system (20 µl) included 10 µl TB Green Premix Ex Taq II (Tli RNaseH Plus, 2×), 0.8 µl PCR Forward Primer (10 µM), 0.8 µl PCR Reverse Primer (10 µM), 0.4 µl ROX Reference Dye (50×), 2 µl DNA template and 6 µl sterile water. The PCR reaction conditions as follows: 95 °C for 30 s followed by 40 cycles at 95 °C for 5 s and 60 °C for 30 s. After the cycling protocol, the final step was applied to all reactions by continuously monitoring fluorescence through the dissociation temperature of the PCR product at a temperature transition rate of 5 °C to generate a melting curve, and then Cooling 50 °C for 30 s. Quantification was conducted according to the 2^−ΔΔct^ method. The relative expression of *CASC11* was analyzed in an Applied Biosystems 7500 Fast real-time PCR system and was normalized with glyceraldehyde-3-phosphate dehydrogenase gene (*GAPDH*). The primers were purchased from Sangon Biotech (Shanghai, China), and the sequences were shown as follows: *CASC11* sense 5′-GCTGCAGA AGGTCCGAAGAA-3′, *CASC11* antisense 5′-TTCACCACGTCCAGTTGCTT-3′; *GAPDH* sense 5′-GGAGCGAGATCCCTCCAAAAT-3, *GAPDH* antisense: 5′-GGCTGTTGTCATACTTCTCATGG-3′.

### Cell transfection

For optimal siRNA transfection efficiency, siRNA sequences were designed to target the human *CASC11* gene and the *CASC11* siRNAs and control siRNAs were obtained from GenePharma (Shanghai GenePharma Co., Ltd., Shanghai, China). The sequences of siRNAs were 5′-GCCCACATCAAGCCTTCAT-3′ (*CASC11* siRNAs) and 5′-UUCUCCGAACGUGUCACGU-3′ (control siRNAs). Cells (2 × 10^5^ cells/well) were added in 6-well plates and transfected with *CASC11* siRNAs (100 nM) or control siRNAs (100 nM) for 48 h using Lipofectamine™ 2000 (Invitrogen; Thermo Fisher scientific, Inc.) according to the instructions.

A *CASC11* overexpression plasmid, pcDNA3.1-CASC11, was commercially constructed by GenePharma (Shanghai, China), and empty pcDNA 3.1 vector (NC) was used as the control. To establish cell lines with transient overexpression of *CASC11*, and CaSki cells (5 × 10^5^/ml) were seeded into 6-well plates and transfected with 10 µg pcDNA3.1-CASC11 plasmid or control pcDNA3.1 vector in medium using lipofectamine™ 2000 (Invitrogen). And the effect of CASC11 silencing and over-expression is shown in Additional file 1: Fig. S1.

### Cell Counting Kit-8 (CCK-8) assay

Cell Counting Kit-8 (CCK-8, Solarbio, China) assay was used to analyze the cell proliferation. Briefly, 100 µl cell suspension were seeded into 96-well plates. 0 h, 12 h, 24 h, 48 h or 96 h after cell transfection, 10 µl CCK-8 reagent was added into the medium incubating in dark at 37 °C for 2 h. Finally, the absorbance at 450 nm was determined with a full wavelength multifunctional enzyme labeling apparatus (TECAN).

### Flow cytometric analysis for apoptosis

48 h after cell transfection, the apoptosis of HeLa cells and CaSki cells was measured with an Annexin V-FITC/PI apoptosis detection kit (BD Biosciences) following the manufacturer’s instructions. The apoptosis rate was analyzed using a FACSCalibur™ flow cytometer (BD Biosciences) as previously mentioned [11].

### Transwell assay for cell invasion and migration

Transwell Chambers (Corning) uncoated or coated with Matrigel were used for assessing the invasion and migration of cervical cancer cells. The detailed methods were as mentioned as Lan described [14]. The migratory or invasive cells was imaged and counted utilizing an optical microscope (Carl Zeiss, Jena, Germany).

### TOP/FOP-Flash luciferase reporter analysis

TOP/FOP-Flash luciferase reporter assay was used to analyze the activity of Wnt/β-catenin signaling pathway. The vector pRL-SV40 was served as the internal reference. Cells in the control groups were co-transfected with pRL-SV40 and TOP/FOP flash (Promega). Cells in NC groups were co-transfected with pRL-SV40, TOP/FOP flash and si-CASC11 NC/pcDNA 3.1. Cells in si-CASC11 group or pcDNA3.1-CASC11 group were co-transfected with pRL-SV40, TOP/FOP flash and si-CASC11 NC/pcDNA 3.1-CASC11. Dual-Luciferase Reporter Assay System (E1910; Promega) was applied to measure the luciferase activity. The ratio of TOP/FOP indicated the activity of Wnt/β-catenin signaling pathway.

### Western blot

Total proteins were extracted from cells with RIPA lysis buffer (P0013B, Beyotime) and nuclear extracts were collected with a Nuclear and Cytoplasmic Protein Extraction Kit (P0027, Beyotime). An enhanced BCA protein assay kit (P0010, Beyotime) was used to measure the protein concentration. 50 μg protein per well was separated by 10% sodium dodecyl sulfate polyacrylamide gel electrophoresis (SDS-PAGE) followed by transferred onto polyvinylidene difluoride membranes (Millipore). After blocked with 5% non-fat milk, the bands were incubated with primary antibodies followed by horseradish peroxidase (HRP)-conjugated secondary antibodies (#7074, 1:2000) from Cell Signaling Technology. The primary antibodies were as follows: β-catenin rabbit monoantibody (#8480, 1:1000), GAPDH rabbit monoantibody (#8884, 1:1000), Lamin B rabbit monoantibody (#12255, 1:1000). The target proteins were visualized with a BeyoECL Plus kit (P0018, Beyotime). Densitometric analysis was carried out with Image J software.

### Statistical analysis

All the experiments were replicated independently at least three times in triplicate. The data were represented as means ± standard deviation (SD) and analyzed with one-way ANOVA using SPSS version 22.0 software (IBM, Chicago, IL, USA). The relationship between the overall survival (OS) of patients with cervical cancer and *CASC11* was evaluated with the Kaplan–Meier test. A value of *p* < 0.05 was considered statistically significant.

## Results

### Up-regulation of *CASCA11* expression in cervical cancer tissues

**Fig. 1:**
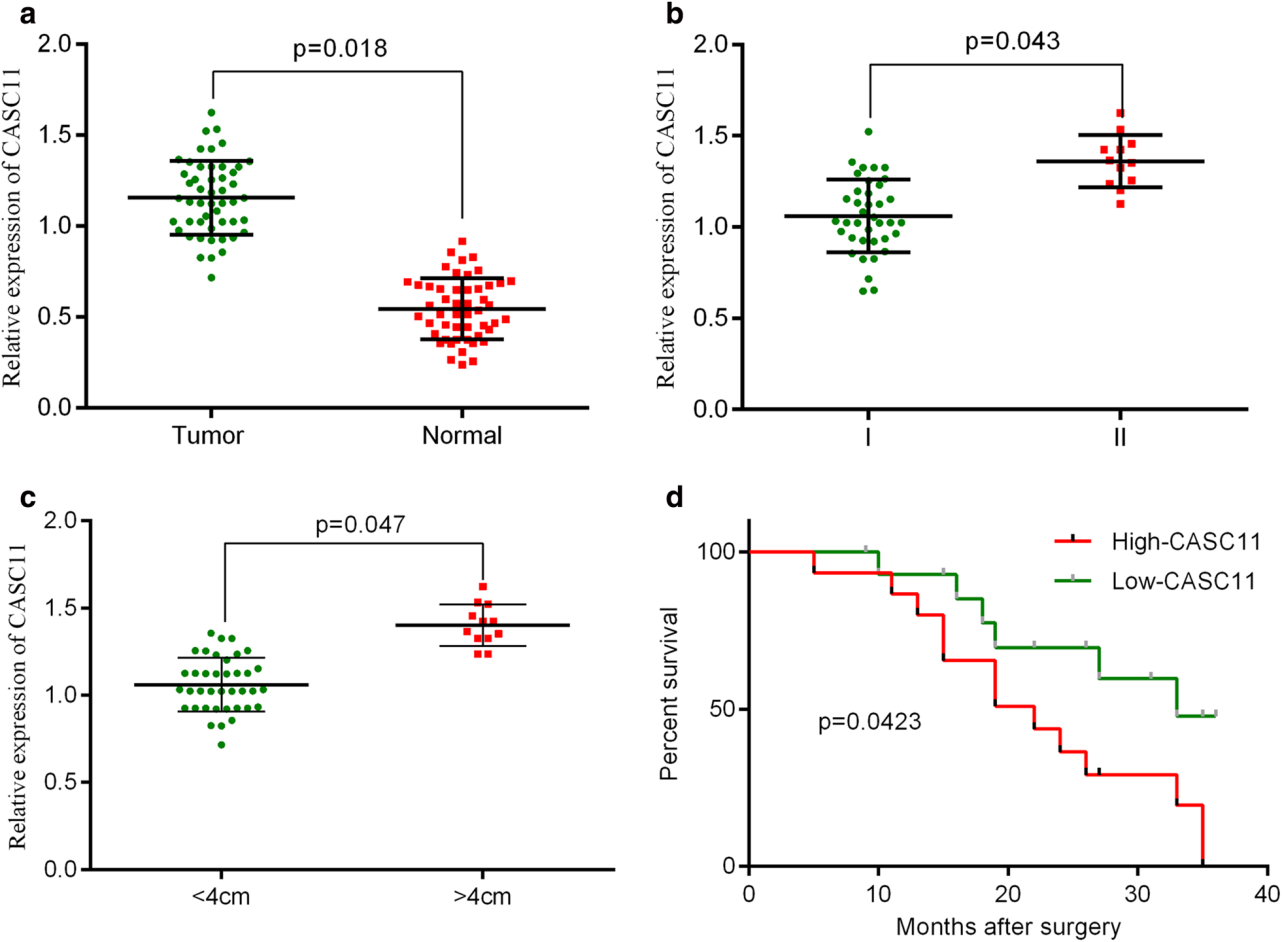
Relative expression levels of lncRNA *CASC11* in cervical cancer tissues. **a** *CASC11* expression was assessed by RT-qPCR assay in cervical cancer samples (n = 50) and adjacent normal samples (n = 50), and *CASC11* was up-regulated in cervical cancer. **b** The expression level of *CASC11* was evaluated by RT-qPCR assay in FIGO stage I (n = 38) and FIGO stage II (n = 12), and *CASC11* was positively related to the FIGO stage. **c** The expression level of *CASC11* was analyzed by RT-qPCR assay in tumor tissues (tumor size < 4 cm, n = 38) and tumor tissues (tumor size ≥ 4 cm, n = 12), and *CASC11* was positively related to the tumor size. **d** Kaplan–Meier test showed that high level of *CASC11* was significantly associated with the low survival rate for cervical cancer patients. *CASC11* expression was detected by RT-qPCR analysis and normalized to *GAPDH* expression (n = 25, respectively). Error bars represent the mean ± SD of 3 independent experiments. *p < 0.5; **p < 0.01

**Fig. 2:**
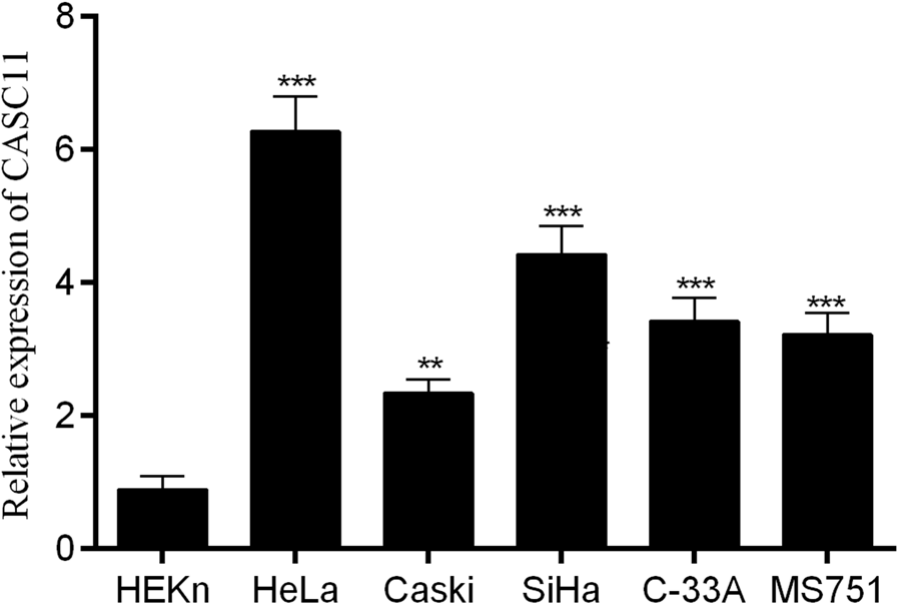
*CASC11* was up-regulated in cervical cancer cell lines. *CASC11* expression was detected by RT-qPCR analysis in HEKn and cervical cancer cell lines (HeLa, Caski, SiHa, C-33A and MS751). The level of *CASC11* was normalized to *GAPDH*. Error bars represent the mean ± SD of 3 independent experiments. **p < 0.01; ***p < 0.001

The relative expression of *CASC11* detected by RT-qPCR was much higher in the tumor samples than that in the adjacent normal tissues (n = 50 tumor; n = 50 adjacent normal; Fig. 1a). The correlation between *CASC11* and clinicopathological characteristics was analyzed in Table 1. The age, menopause, lymph node metastasis and lymphatic vascular space invasion of patients have little effect on *CASC11* expression, but which was positively correlated with the FIGO stage and the tumor size (Fig. 1b, c). 50 cervical cancer patients were divided into high and low *CASC11* expression groups (n = 25, respectively) according to the median *CASC11* expression level. Kaplan–Meier test showed that high level of *CASC11* was significantly associated with the low survival rate for cervical cancer patients (Fig. 1d). We further measured the RNA levels of *CASC11* in cervical cancer cells finding that the relative expression of *CASC11* in HeLa, CaSki, SiHa, C-33A and MS751 were remarkably higher than that in the normal ectocervical cells HEKn. Furthermore, among the cervical cancer cell lines, HeLa cells showed the highest expression of *CASC11* and CaSki cells displayed the lowest *CASC11* level (Fig. 2). So, we chose HeLa cells for si-CASC11 transfecting and chose CaSki cells for pcDNA3.1-CASC11 transfection.

**Table 1:** Correlation between CASC11 and clinicopathological characteristics (n = 50)

Clinicopathologic feature	N (%)	CASC11 (mean ± SEM)	*p*-value
Age			0.836
≤ 60	19 (38%)	1.23 ± 0.17	
> 60	31 (62%)	1.17 ± 0.20	
Tumor size (cm)			0.047*
< 4	38 (76%)	1.05 ± 0.12	
≥ 4	12 (24%)	1.42 ± 0.15	
Menopause			
Yes	26 (52%)	1.29 ± 0.17	0.925
No	24 (48%)	1.30 ± 0.14	
Lymph node metastasis			
Negative	23 (46%)	1.25 ± 0.20	0.918
Positive	27 (54%)	1.27 ± 0.18	
Lymphativ vascular space invasion			
Negative	29 (58%)	1.23 ± 0.12	0.816
Positive	21 (42%)	1.36 ± 0.10	
FIGO stage			0.043*
I	38 (78%)	1.03 ± 0.15	
II	12 (24%)	1.39 ± 0.16	

*p < 0.5

### *CASC11* promoted the proliferation and inhibited the apoptosis of cervical cancer cells

**Fig. 3:**
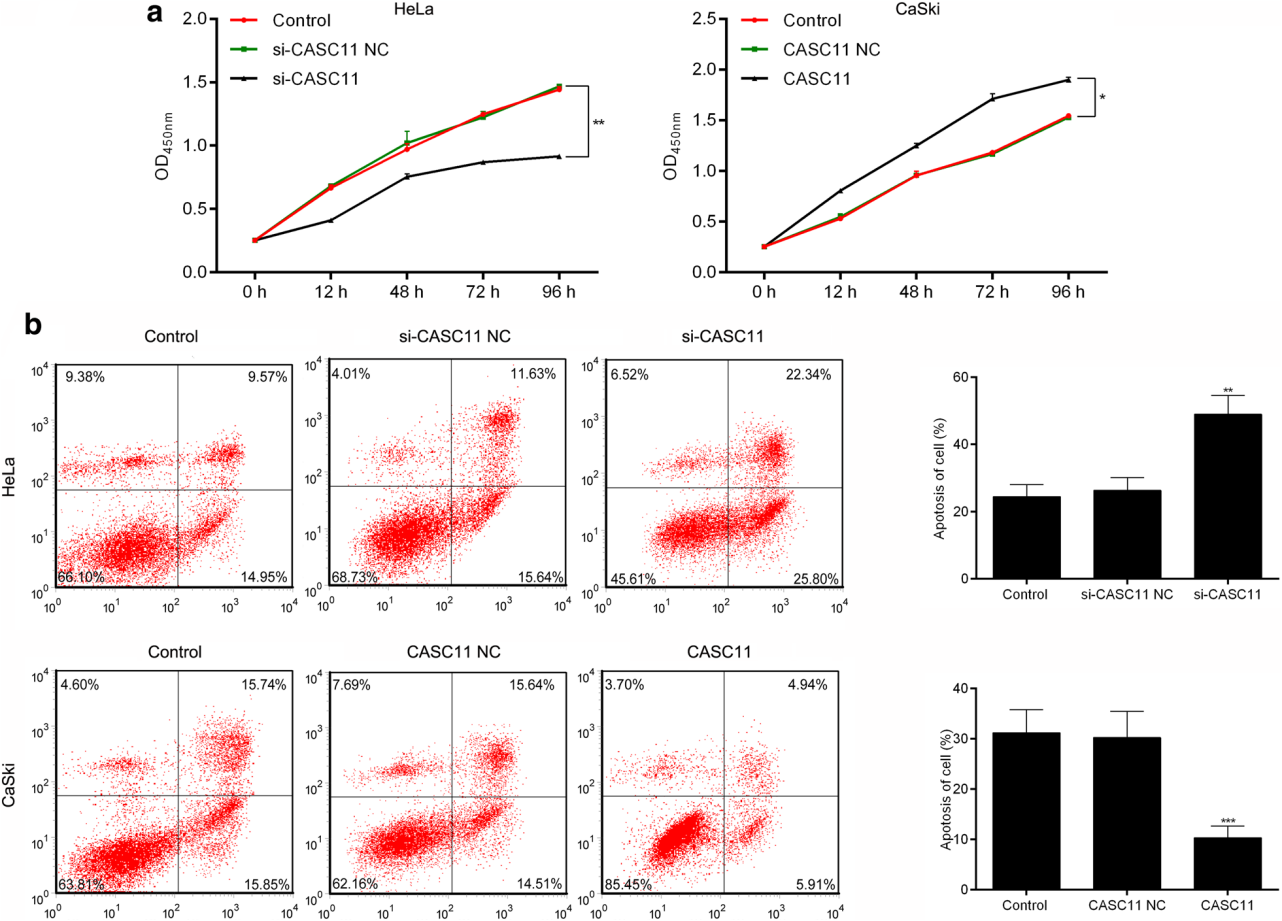
*CASC11* promoted the proliferation and inhibited the apoptosis in cervical cancer cells. **a** Cell proliferation was evaluated by CCK8 assay, and silence of *CASC11* inhibited the proliferation of HeLa cells and up-regulating CASC11 promoted the proliferation of CaSki cells. **b** Flow cytometry showed that silence of *CASC11* promoted the apoptosis of HeLa cells and the up-regulation of *CASC11* suppressed the apoptosis of CaSki cells. Data were shown as the mean ± SD of 3 independent experiments. *p < 0.5; **p < 0.01; ***p < 0.001

Silencing *CASC11* with siRNA in HeLa cells significantly decreased the cell proliferation and increased the apoptosis of cells compared to that in the control cells and the si-CASC11 NC-transfected cells. Conversely, overexpression of *CASC11* by transfecting the CaSki cells with pcDNA3.1-CASC11 significantly increased the cell proliferation while decreased the apoptosis of cells versus that in control and *CASC11* NC groups (Fig. 3a, b).

### *CASC11* promoted the migration and invasion of cervical cancer cells.

**Fig. 4:**
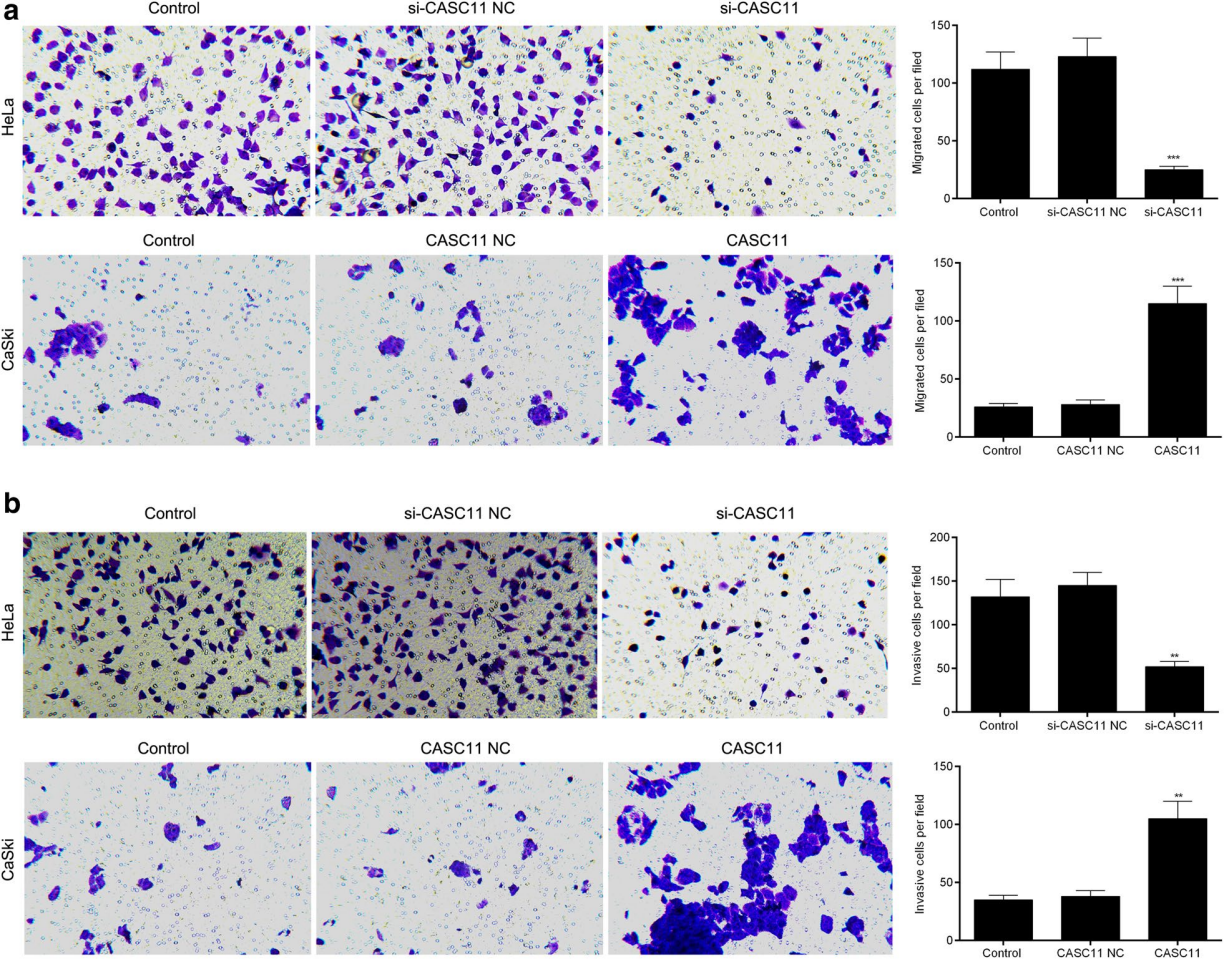
*CASC11* promoted the migration and invasion in cervical cancer cells. **a** Transwell assay showed that silencing *CASC11* inhibited the migration of HeLa cells and up-regulating *CASC11* promoted the migration of CaSki cells. **b** Cell invasion was measured by Transwell assay, and silencing *CASC11* inhibited the invasion of HeLa cells and up-regulating *CASC11* promoted the invasion of CaSki cells. Data were shown as the mean ± SD of 3 independent experiments. **p < 0.01; ***p < 0.001

Transwell experiments showed that knockdown of *CASC11* in HeLa cells showed significantly lower abilities of migration and invasion than that in control and si-CASC11 NC-transfected cells while overexpression of *CASC11* in CaSki cells displayed much higher abilities of migration and invasion than that in control and *CASC11* NC groups (Fig. 4a, b).

### *CASC11* involved in the activating of Wnt/β-catenin signaling pathway

**Fig. 5:**
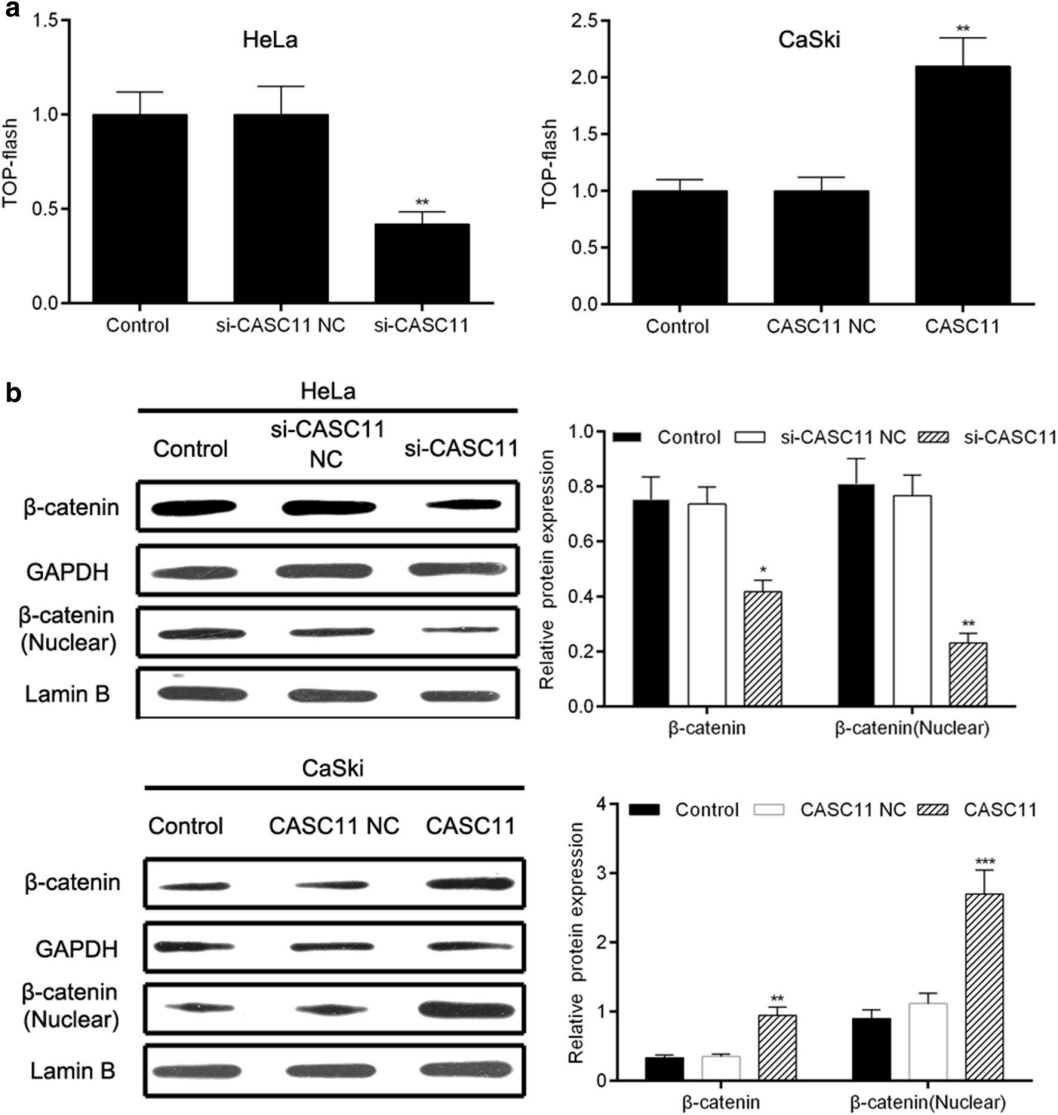
*CASC11* involved in the activating of Wnt/β-catenin signaling pathway. **a** TOP/FOP-Flash luciferase reporter assay was used to analyze the effect of *CASC11* on the activity of Wnt/β-catenin signaling pathway in HeLa cells and CaSki cells. **b** Western blot analysis was used to evaluate the expressions of total and nuclear β-catenin in si-CASC-transfected HeLa cells and pcDNA 3.1-CASC11-transfected CaSki cells. Data were shown as the mean ± SD of 3 independent experiments. *p < 0.5; **p < 0.01; ***p < 0.001

TOP/FOP-Flash luciferase reporter assay showed that down-regulated the expression of *CASC11* in HeLa cells inhibited the activity of Wnt/β-catenin signaling pathway while overexpression of *CASC11* in CaSki cells significantly up-regulated the signaling activity (Fig. 5a). Western blot analysis indicated that silencing *CASC11* significantly decreased the total and nuclear expressions of β-catenin, while overexpression of *CASC11* observably increased the total and nuclear expressions of β-catenin (Fig. 5b). These results suggested that *CASC11* was involved in the activation of Wnt/β-catenin signaling pathway.

### *CASC11* promoted the migration and invasion of cervical cancer cells through activating Wnt/β-catenin signaling

**Fig. 6:**
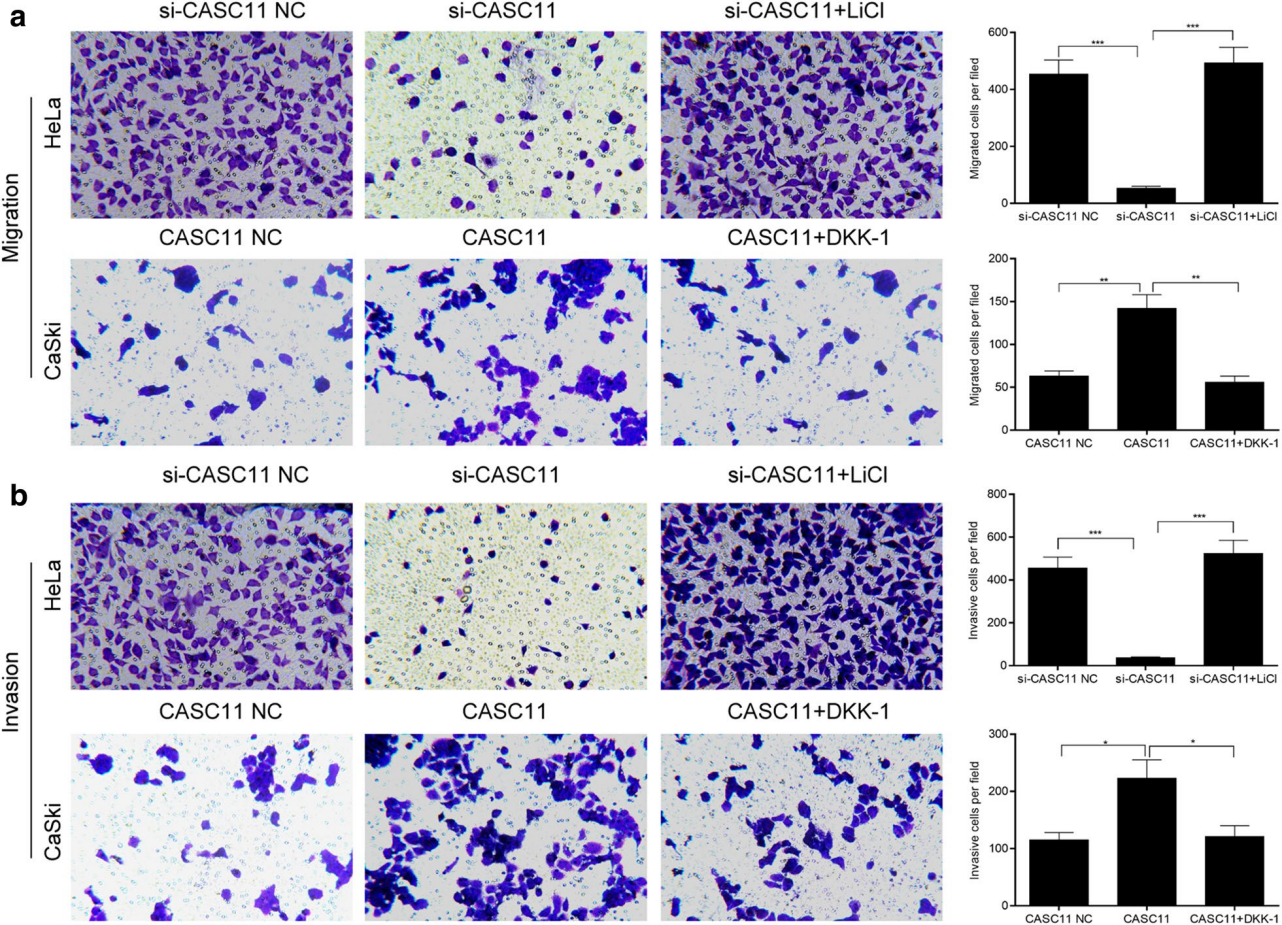
*CASC11* promoted the migration and invasion in cervical cancer cells through activating Wnt/β-catenin signaling. HeLa cells were transfected with si-CASC11 or negative control (NC), and treated with LiCl, respectively; HeLa cells were transfected with pcDNA 3.1-CASC11 or control, and treated with Dkk-1, respectively. **a** Cell migration was examined by Transwell assay, and knockdown of CASC11 inhibited the migration of cells, which could be reserved by LiCl; overexpression of CASC11 promoted the migration of cells, which could be reserved by Dkk-1. **b** Cell invasion was examined by Transwell assay, and knockdown of CASC11 inhibited the invasion of cells, which could be reserved by LiCl; overexpression of CASC11 promoted the invasion of cells, which could be reserved by Dkk-1. 5 fields was taken with an optical microscope. Data were shown as the mean ± SD of 3 independent experiments. *p < 0.5; **p < 0.01; ***p < 0.001

**Fig. 7:**
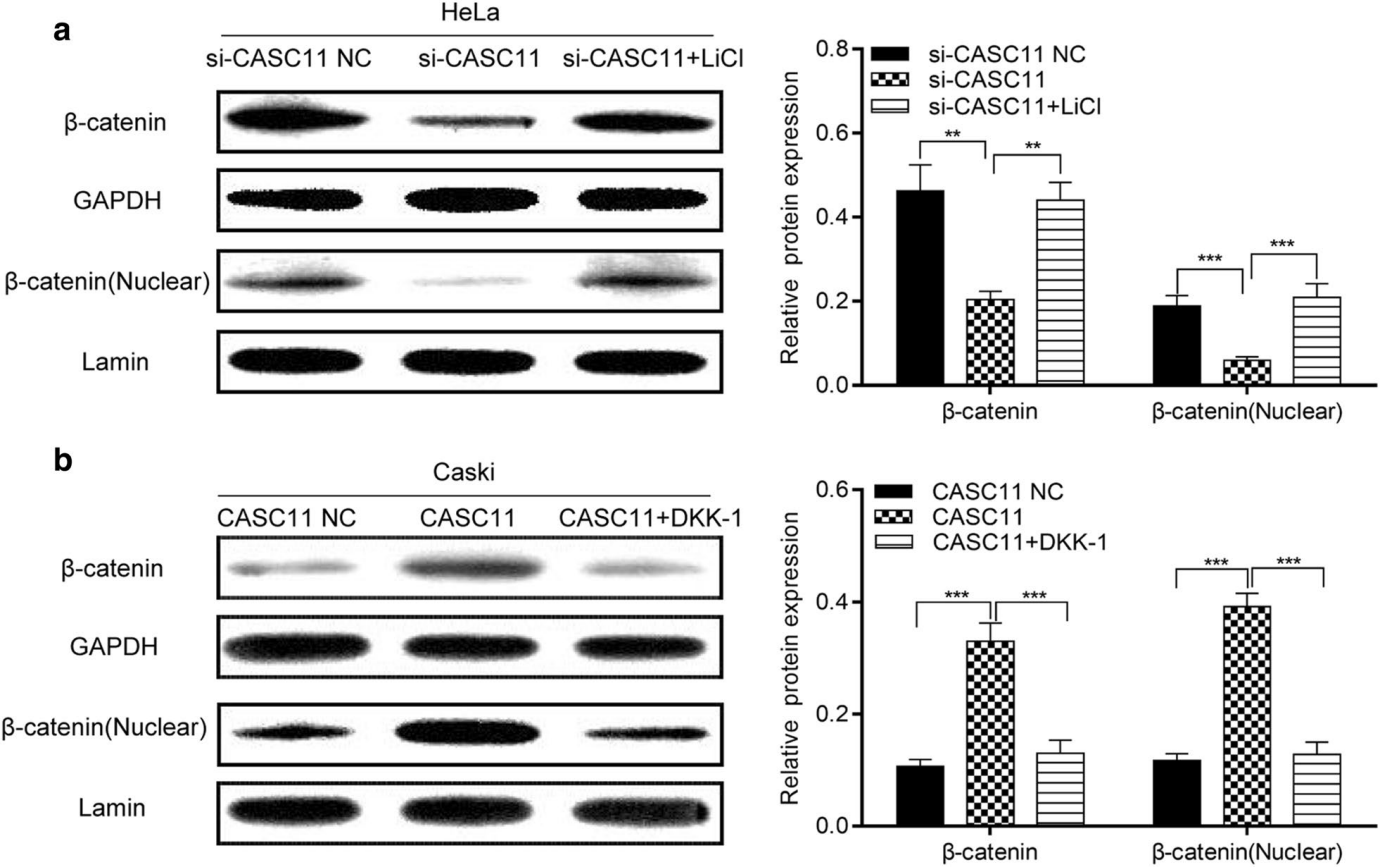
The effects of LiCl and Dkk-1 on the expression of CASC11 in HeLa and CaSki cells. **a** HeLa cells were transfected with si-CASC11 or NC, and treated with LiCl, respectively. The expression level of β-catenin was measured by western blot assay. **b** HeLa cells were transfected with pcDNA 3.1-CASC11 or control, and treated with Dkk-1, respectively. β-Catenin expression was analyzed by western blot assay. Data were shown as the mean ± SD of 3 independent experiments. **p < 0.01; ***p < 0.001

As shown as Fig. 6a, b, the si-CASC11-transfected cells treated with the Wnt signaling activator LiCl or si-CASC11 NC showed a much higher ability of migration and invasion than that without LiCl treatment. The pcDNA3.1-CASC11 transfected cells treated with the Wnt signaling inhibitor Dkk-1 or CASC11 NC displayed a lower ability of migration and invasion than that without Dkk-1 treatment. Western blot displayed that compared to the cell only transfected with si-CASC11 or pcDNA3.1-CASC11, LiCl significantly increased the expressions of total and nuclear β-catenin and Dkk-1 significantly decreased the total and nuclear β-catenin expressions (Fig. 7a, b). Therefore, these results indicated that *CASC11* requires an active Wnt/β-catenin pathway to contribute to the phenotype.

### Knock-down *CASC11* inhibited the proliferation of tumor in vivo

**Fig. 8:**
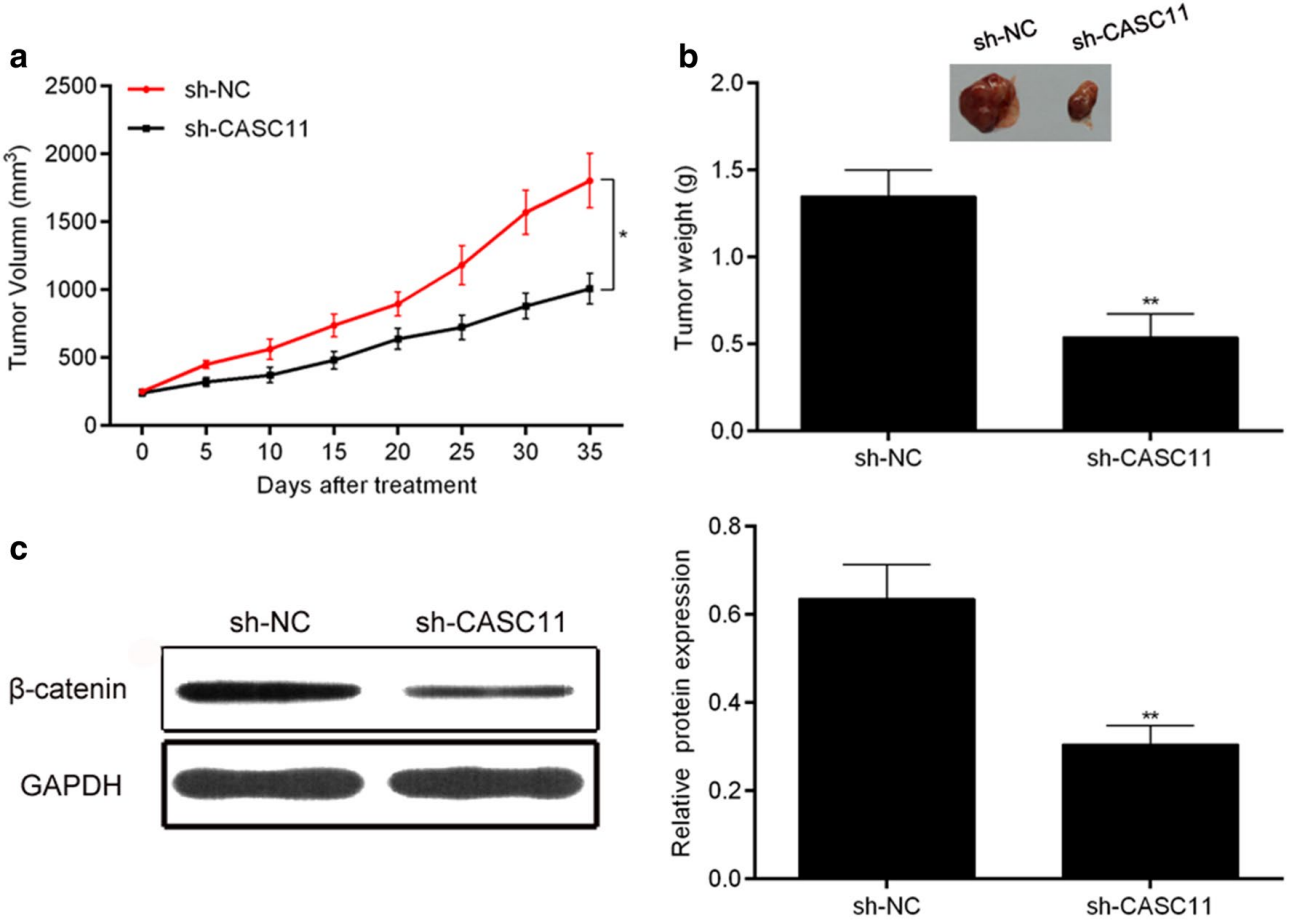
Knock-down *CASC11* inhibited tumor proliferation in vivo*.* HeLa cells were transfected with sh-CASC11 or sh-NC, respectively. And the cells were injected subcutaneously into nude mice. **a** Tumor volume was measured, and silencing *CASC11* decreased the tumor volume compared to the siRNA NC group.** b** Tumor weight was measured, and knockdown *CASC11* inhibited tumor formation in nude mice (n = 6 each group). **c** β-Catenin expression was assessed by western blot assay in the si-NC mice and in the si-CASC11 mice. Data were shown as the mean ± SD of 3 independent experiments. **p < 0.01

As shown as Fig. 8a, b, the tumor volume and weight of mice in the HeLa/si-CASC11 group were bigger and heavier than that in the HeLa/si-CASC11NC group. As expected, western blot analysis showed that the expression of β-catenin in the HeLa/si-CASC11 group was much higher than that in the HeLa/si-CASC11 group (Fig. 8c). These data hints that *CASC11* promoted the proliferation of tumor in vivo*.*

## Discussion

Chromosome 8q24, which is about 600 kb in length, was once thought to be a gene desert. Genome-wide association study (GWAS) has shown that genetic mutations in 8q24 region are associated with the risk of prostate cancer, colorectal cancer and other malignant tumors [15]. Another study indicated that *MYC* (8q24) played a vital part in the progression of CIN to cervical cancer [3]. Recently, Shen et al. [16] found that in HeLa cell line, the integrated HPV, MYC and 8q24.22 was close to each other, which might form a trimer in spatial location promoting the development of cervical cancer. *CASC11* is a newly discovered lncRNA ~ 2.1 kb upstream of c-Myc in chromosome 8q24 gene desert. The upregulation of *CASC11* was reported to participated in the proliferation, migration and invasion of tumor cells in hepatic carcinoma, gastric and colorectal cancer. Zhang et al. [11] found that c-Myc could directly bind to the *CASC11* promoter regions increasing the promoter histone acetylation to enhance *CASC11* expression in colorectal cancer. Therefore, we hypothesized that *CASC11* has a similar function for cervical cancer and discovered that *CASC11* promoted the proliferation, metastasis and invasion of cervical cancer cells through Wnt**/**β-catenin signaling pathway.

Because of the high recurrence rate, poor prognosis and current dissatisfactory molecular biomarkers that can predict the prognosis of cervical cancer patients, it is urgent to find new diagnostic and prognostic biomarkers of cervical cancer. In recent years, more and more cervical cancer-related lncRNAs were discovered providing new insights for the treatment of cervical cancer. The lncRNA distal-less homeobox 6 antisense 1 (*DLX6-AS1*) was reported to be markedly upregulated in cervical cancer tissues and cell lines. Knocking out of *DLX6-AS1* inhibited the cell proliferation and induced cell apoptosis by targeting to miR199a [17]. Wang et al. [18] found that *BLACAT1* (also known as linc-UBC1) may be served as a novel prognostic biomarker for its positive effects on promoting the proliferation, migration and invasion of cervical cancer cells. However, some lncRNA expressions, for example, lncRNA *ZNF667-AS1*, have been proved experimentally to be negatively associated with the tumor volumes, FIGO staging and positively related to the patients’ survival rate. Overexpression of *ZNF667-AS1* significantly decreased the proliferation ability and cell cloning ability of HeLa cells [19]. In the present study, we discovered that *CASC11*, the expression of which was positively associated with the tumor size and the FIGO staging, was up-regulated in the cervical cancer tissues and cell lines. Kaplan–Meier test showed *CASC11* expression was negatively related to the cervical cancer patients’ survival rate. Silencing *CASC11* by siRNA technology inhibited the proliferation, migration as well as invasion and promoted the cell apoptosis. Conversely, overexpression of *CASC11* facilitated the cancer cell growth and suppressed the apoptosis.

Studies have shown that abnormal expressions of signaling molecules including phosphatidylinositol 3-kinase (PI3K), epidermal growth factor receptor (EGF-R), β-catenin, extracellular signal-regulated kinase (ERK) and Bcl-2 played significant parts in cervical cancer progression [20]. Wnt/β-catenin is a canonical pathway that can participate in cell growth, regulation and differentiation. Under normal conditions, β-catenin is phosphorylated keeping at a low cytoplasmic level at the presence of a GSK3β multi-protein complex. When the signaling pathway is activated, GSK3β kept as an inactive state, which facilitates the cytoplasmic β-catenin to transfer into the nucleus regulating the expressions of target genes [21]. Many lncRNAs take part in various types cancers through regulating Wnt/β-catenin signaling pathway, like liver cancer, colorectal cancer, breast tumor and so on [5]. Likewise, lncRNAs, such as *BLACAT1*, *LINC00675* and *TUG1*, promoted the cervical cancer progression by activating Wnt/β-catenin signaling pathway [18, 22, 23]. In our research, we found si-CASC11 decreased the activity of Wnt/β-catenin signaling pathway and the expression of β-catenin while treatment the si-CASC11-transfected cells with LiCl significantly promoted the migration and invasion of HeLa cells by up-regulating the expression of β-catenin. Conversely, overexpression of *CASC11* increased the activity of Wnt/β-catenin signaling pathway and the expression of β-catenin while treatment the pcDNA3.1-CASC11-transfected cells with Dkk-1 significantly depressed the migration and invasion of CaSki cells by reducing the expression of β-catenin. Effects of CASC11 downregulation and overexpression on migration and invasion and in vivo studies should include the determination of animal metastasis, which should be the focus of future studies. Tumor formation in nude mice showed that inhibition of *CASC11* expression decreased the tumor volumes and weight by reducing the expression of β-catenin. These results indicated that *CASC11* promoted the cervical cancer progression by activating Wnt/β-catenin signaling pathway.

## Conclusion

Our study demonstrated that *CASC11* promoted the cervical cancer progression by activating Wnt/β-catenin signaling pathway for the first time, which provides a new target or a potential diagnostic biomarker of the treatment for cervical cancer. As known to all, lncRNAs function in cancers by interacting with miRNAs, mRNAs and proteins. Therefore, next we will investigate the direct targets of *CASC11* in cervical cancers and explore its mechanism comprehensively. In addition, the specific regulatory mechanism of CASC11 on Wnt signaling needs further study in the future.

## Additional file

**Additional file 1: Figure S1.** Expression levels of lncRNA *CASC11* were determined by RT-qPCR after silencing of si-CASC11-NC, si-CASC11, or overexpression of CASC11-NC, CASC11. Values indicate each point and the average. Values are mean ± SEM Statistical significance was assessed by the Student's *t* test. Differences were considered statistically significant. *p < 0.05; ***p < 0.001 (n = 3 per group).

## Data Availability

Source data and material will be made available upon request.

## Abbreviations

*CASC11*: cancer susceptibility candidate 11
lncRNA: long non-coding RNA
CIN: cervical intraepithelial neoplasia
HPV: human papillomavirus
HOTAIR: HOX transcript antisense RNA
MALAT1: metastasis associated lung adenocarcinoma transcript 1
hnRNP-K: heterogeneous ribonucleoprotein K

## References

[1] Walboomers JM, Jacobs MV, Manos MM, Bosch FX, Kummer JA, Shah KV (1999). Human papillomavirus is a necessary cause of invasive cervical cancer worldwide. J Pathol..

[2] Burd EM (2003). Human papillomavirus and cervical cancer. Clin Microbiol Rev..

[3] Kuglik P, Kasikova K, Smetana J, Vallova V, Lastuvkova A, Moukova L (2015). Molecular cytogenetic analyses of hTERC (3q26) and MYC (8q24) genes amplifications in correlation with oncogenic human papillomavirus infection in Czech patients with cervical intraepithelial neoplasia and cervical carcinomas. Neoplasma..

[4] Sharma S, Mandal P, Sadhukhan T, Chowdhury RR, Mondal NR, Chakravarty B (2015). Bridging links between long noncoding RNA HOTAIR and HPV oncoprotein E7 in cervical cancer pathogenesis. Scientific Rep..

[5] Hu X-Y, Hou P-F, Li T-T, Quan H-Y, Li M-L, Lin T (2018). The roles of Wnt/β-catenin signaling pathway related lncRNAs in cancer. Int J Biol Sci..

[6] Chandra Gupta S, Nandan Tripathi Y (2017). Potential of long non-coding RNAs in cancer patients: from biomarkers to therapeutic targets. Int J Cancer..

[7] Dong J, Su M, Chang W, Zhang K, Wu S, Xu T (2017). Long non-coding RNAs on the stage of cervical cancer. Oncol Rep..

[8] Xin Y, Li Z, Zheng H, Chan MT, Ka K, Wu W (2017). CCAT 2: a novel oncogenic long non-coding RNA in human cancers. Cell Prolif..

[9] Zhang Z, Zhou C, Chang Y, Zhang Z, Hu Y, Zhang F (2016). Long non-coding RNA CASC11 interacts with hnRNP-K and activates the WNT/β-catenin pathway to promote growth and metastasis in colorectal cancer. Cancer Lett..

[10] Han Y, Chen M, Wang A, Fan X (2019). STAT3-induced upregulation of lncRNA CASC11 promotes the cell migration, invasion and epithelial–mesenchymal transition in hepatocellular carcinoma by epigenetically silencing PTEN and activating PI3K/AKT signaling pathway. Biochem Biophys Res Commun..

[11] Zhang L, Kang W, Lu X, Ma S, Dong L, Zou B (2018). LncRNA CASC11 promoted gastric cancer cell proliferation, migration and invasion in vitro by regulating cell cycle pathway. Cell Cycle.

[12] Ayala-Calvillo E, Mojica-Vázquez LH, García-Carrancá A, González-Maya L (2018). Wnt/β-catenin pathway activation and silencing of the APC gene in HPV-positive human cervical cancer-derived cells. Mol Med Rep..

[13] Peng J, Hou F, Feng J, Xu SX, Meng XY (2018). Long non-coding RNA BCYRN1 promotes the proliferation and metastasis of cervical cancer via targeting microRNA-138 in vitro and in vivo. Oncol Lett..

[14] Lan K, Zhao Y, Fan Y, Ma B, Yang S, Liu Q (2017). Sulfiredoxin may promote cervical cancer metastasis via Wnt/β-catenin signaling pathway. Int J Mol Sci..

[15] Ghoussaini M, Song H, Koessler T, Al Olama AA, Kote-Jarai Z, Driver KE (2008). Multiple loci with different cancer specificities within the 8q24 gene desert. JNCI J Natl Cancer Inst..

[16] Shen C, Liu Y, Shi S, Zhang R, Zhang T, Xu Q (2017). Long-distance interaction of the integrated HPV fragment with MYC gene and 8q24 22 region upregulating the allele-specific MYC expression in HeLa cells. Int J Cancer..

[17] Wang X, Lin Y, Liu J (2018). Long non-coding RNA DLX6-AS1 promotes proliferation by acting as a ceRNA targeting miR-199a in cervical cancer. Mol Med Rep..

[18] Wang C, Li Y, Tian H, Bao X, Wang Z (2018). Long non-coding RNA BLACAT1 promotes cell proliferation, migration and invasion in cervical cancer through activation of Wnt/β-catenin signaling pathway. Eur Rev Med Pharmacol Sci..

[19] Zhao L, Li R, Han D, Zhang X, Nian G, Wu M (2017). Independent prognostic Factor of low-expressed LncRNA ZNF667-AS1 for cervical cancer and inhibitory function on the proliferation of cervical cancer. Eur Rev Med Pharmacol Sci..

[20] Yang M, Wang M, Li X, Xie Y, Xia X, Tian J (2018). The role of lncRNAs in signaling pathway implicated in CC. J Cell Biochem..

[21] Duchartre Y, Kim Y-M, Kahn M (2016). The Wnt signaling pathway in cancer. Crit Rev Oncol Hematol..

[22] Ma S, Deng X, Yang Y, Zhang Q, Zhou T, Liu Z (2018). The lncRNA LINC00675 regulates cell proliferation, migration, and invasion by affecting Wnt/β-catenin signaling in cervical cancer. Biomed Pharmacother..

[23] Zhu J, Shi H, Liu H, Wang X, Li F (2017). Long non-coding RNA TUG1 promotes cervical cancer progression by regulating the miR-138-5p-SIRT1 axis. Oncotarget..

