# Supplementary figures and images for: LncRNA CASC11 promotes the cervical cancer progression by activating Wnt/beta-catenin signaling pathway
Source: Biol Res. 2019 Jun 29;52:33. doi: 10.1186/s40659-019-0240-9 (PMC6599525; doi:10.1186/s40659-019-0240-9)

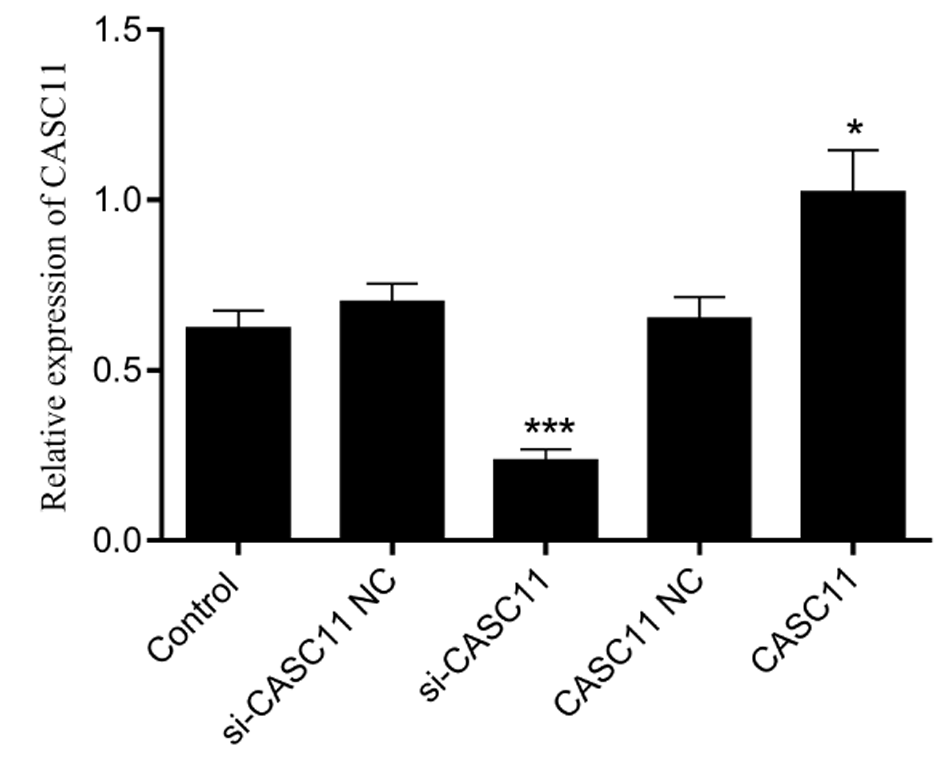

Supplement: Supplementary file 1 — Additional file 1: Figure S1. Expression levels of lncRNA CASC11 were determined by RT-qPCR after silencing of si-CASC11-NC, si-CASC11, or overexpression of CASC11-NC, CASC11. Values indicate each point and the average. Values are mean ± SEM Statistical significance was assessed by the Student's t test. Differences were considered statistically significant. *p < 0.05; ***p < 0.001 (n = 3 per group). [file 40659_2019_240_MOESM1_ESM.tif]
